# Toward Mass Spectrometry Imaging in the Metabolomics Scale: Increasing Metabolic Coverage Through Multiple On-Tissue Chemical Modifications

**DOI:** 10.3389/fpls.2019.00860

**Published:** 2019-07-12

**Authors:** Maria Emilia Dueñas, Evan A. Larson, Young Jin Lee

**Affiliations:** Department of Chemistry, Iowa State University, Ames, IA, United States

**Keywords:** mass spectrometry imaging, metabolomics, on-tissue derivatization, high-spatial resolution, maize, single cell

## Abstract

Exploring the metabolic differences directly on tissues is essential for the comprehensive understanding of how multicellular organisms function. Mass spectrometry imaging (MSI) is an attractive technique toward this goal; however, MSI in metabolomics scale has been hindered by multiple limitations. This is most notable for single cell level high-spatial resolution imaging because of the limited number of molecules in small sampling size and the low ionization yields of many metabolites. Several on-tissue chemical derivatization approaches have been reported to increase MSI signals of targeted compounds, especially in matrix-assisted laser desorption/ionization (MALDI)-MSI. Herein, we adopt a combination of chemical derivatization reactions, to selectively enhance metabolite signals of a specific functional group for each consecutive tissue section. Three well-known on-tissue derivatization methods were used as a proof of concept experiment: coniferyl aldehyde for primary amines, Girard’s reagent T for carbonyl groups, and 2-picolylamine for carboxylic acids. This strategy was applied to the cross-sections of leaves and roots from two different maize genotypes (B73 and Mo17), and enabled the detection of over six hundred new unique metabolite features compared to without modification. Statistical analysis indicated quantitative variation between metabolites in the tissue sections, while MS images revealed differences in localization of these metabolites. Combined, this untargeted approach facilitated the visualization of various classes of compounds, demonstrating the potential for untargeted MSI in the metabolomics scale.

## Introduction

Modern metabolomics technology, led by innovations in mass spectrometry, has allowed for systems biological understanding at the gene, RNA, protein, or metabolite level (Zhang et al., 2012). Most of these studies, however, are performed ignoring the variations between different cell types or even different tissue types. As multicellular organisms, plants and animals achieve their complex living activities through the highly organized metabolic interplay of a three-dimensional array of individual cells and tissues. Thus, understanding the metabolic differences of individual cells directly on tissue is essential for the comprehensive understanding of how multicellular organisms function.

Mass spectrometry imaging (MSI), especially matrix-assisted laser desorption/ionization (MALDI)-MSI, is an attractive technique toward this goal due to its soft ionization and small sampling size. Recently, MS images with pixel sizes from 1 to 5 μm have been obtained (Zavalin et al., 2013, 2014; Korte et al., 2015; Kompauer et al., 2016) which allowed to study the fine details of molecular distribution at the cellular and sub-cellular levels. The Caprioli group has achieved spatial resolution between 0.5 and 5 μm on a routine basis by modifying commercial instruments with custom optical and mechanical systems. The laser can be optimized by reducing optical aberrations, expanding the beam prior to focusing, and by reducing the focal length (Zavalin et al., 2013, 2014). Moreover, the laser beam can be irradiated from the back side of the sample to achieve molecular images down to 1 μm spatial resolution (Zavalin et al., 2012; Thiery-Lavenant et al., 2013). Sub-cellular imaging capabilities have also been achieved using AP-SMALDI. The Spengler group achieved lateral resolution down to 1.4–5 μm (Kompauer et al., 2016; Khalil et al., 2017), which visualized the subcellular localization of lipids, metabolites, and peptides. Furthermore, tg-LAESI MS and nano-DESI have been used, with a spot size of 10–20 μm, for *in situ* analysis of small adherent cell populations (Jacobson et al., 2015) and for imaging of pancreatic islets (Yin et al., 2018).

Our group has established 5–10 μm high-spatial resolution with MALDI-ion trap-Orbitrap and has applied this platform to visualize the distribution of a number of different metabolites in maize leaves (Korte et al., 2015; Dueñas et al., 2017), seeds (Dueñas et al., 2016; Feenstra et al., 2017a), and roots (Feenstra et al., 2017b). Previously, the work of Korte et al. (2015) revealed that molecular distribution of metabolites and lipids may be heterogeneous even among cells of the same tissue type. More recently, this platform was applied to explore the quantitative fatty acyl distributions of thylakoid membrane lipids along the developmental gradient of maize leaves for two inbred lines and their hybrids of maize (Dueñas et al., 2017). This study demonstrated that certain thylakoid membrane lipids show genotype-specific differences in cellular distribution. Additionally, this platform has recently demonstrated the subcellular localization of Arabidopside A to the chloroplast (Hansen et al., 2018b).

In terms of metabolic coverage, however, MSI has been mostly limited to a few targeted metabolites due to several obstacles including the lack of chromatographic separation, matrix-dependent analyte selectively, and the limited number of molecules available in a small sampling size. Recently, Feenstra et al. (2015) proposed a MSI methodology that combined multiplex MSI data acquisition with several different matrices on consecutive tissue sections. Even though this approach increased the metabolic coverage by using matrix-dependent selectivity of analytes, the number of metabolites that could be visualized is still critically limited for those metabolites with low ionization efficiencies.

On-tissue chemical modifications have been suggested as an alternative approach to improve the ionization efficiency of targeted compounds in MSI. Manier et al. (2014) used 4-hydroxy-3-methoxycinnamaldehyde (coniferyl aldehyde; CA) to derivatize primary amine group in amino acids and neurotransmitters on pig adrenal gland tissues for MALDI-MSI. Pyrylium salt and *N*-hydroxysuccinimidyl carbamate have also been used to derivatize primary amine groups (Toue et al., 2014; Shariatgorji et al., 2015). GT, a quaternary ammonium acetylhydrazine chloride, has been used to chemical modify carbonyl groups on-tissue, specifically corticosteroids in rat adrenal and mouse sections (Shimma et al., 2016), testosterone in mouse testis (Cobice et al., 2016), and triamcinolone acetonide in human cartilage (Barré et al., 2016). As another on-tissue chemical modification, 2-PA was used to visualize endogenous fatty acids in rat brain tissues (Wu et al., 2016). All of these reactions proceed quickly at room temperature without the need of additional of specific buffers.

In this work, multiple chemical derivatization is used to selectively enhance the metabolite signals for a sub-metabolome at a time. This is accomplished by performing on-tissue derivatization using three known chemical reactions: CA for primary amines (Manier et al., 2014), GT for carbonyl groups (Barré et al., 2016), and 2-PA for carboxylic acids (Wu et al., 2016). These reactions, previously used for targeted analysis of a few selected compounds, are used in combination enabling the visualization of different classes of compounds in an untargeted manner. In a proof of concept application, this multiple on-tissue derivatization strategy is applied to explore the differences in metabolite coverage in roots and leaves of two different maize genotypes (B73 and Mo17).

## Experimental

### Materials

Methanol, water, acetonitrile were purchased from Millipore-Sigma (St. Louis, MO, United States) in CHROMASOLV LC-MS or Plus grade. GT, CA, 2-PA, DPDS, TPP, DAN, DHB, and potassium acetate were purchased from Millipore-Sigma. Gelatin from porcine skin (300 bloom) was purchased from Electron Microscopy Sciences (Hatfield, PA, United States). Gold sputter targets were purchased from Ted Pella Inc. (Redding, CA, United States). B73 and Mo17 inbred maize seeds were obtained from Dr. Marna Yandeau-Nelson at Iowa State University.

### Maize Tissue Growth and Harvest

Procedures for maize root growth is described in detail elsewhere (Feenstra et al., 2017b). Briefly, a row of seeds, with the embryo facing down, were staggered along the top edge of a moist brown paper towel. The paper towel was rolled tight enough to hold the seeds in place and the bottom of the roll was placed into a 1 L beaker half filled with water. The beaker was placed in the dark and the seeds were allowed to grow for 10 days. The beaker was monitored periodically to ensure enough water remained inside in order to submerge the lower portion of the paper towel roll. The roots were harvested, 10 days after planting, when the lengths of the primary root was 10–14 cm, as measured from the tip of the root. The area of interest was embedded in a 10% (w/v) gelatin solution in a cryo-mold and flash-frozen in liquid nitrogen until the gelatin was ∼80% solidified.

For maize leaf imaging, maize seeds were planted in soil and grown in a climate-controlled greenhouse at 30% humidity under a diurnal cycle of 16 h of light and 8 h of dark at 27°C and 24°C, respectively. Plant seedlings were harvested 11 days after planting. The sections of leaves were collected at the midpoint of leaf 3. The fresh maize leaf sections were embedded in gelatin before cryo-sectioning.

### Sample Preparation

The molds were transferred to a cryostat (CM 1850, Leica Microsystems, Buffalo Grove, IL, United States) pre-chilled to −20°C, and allowed to thermally equilibrate for 30 min. Tissue samples were cryo-sectioned at 10 μm thickness, collected with Cryo-Jane tape (Leica Biosystems), and attached to a pre-chilled glass slide. The prepared slides were placed onto a chilled aluminum block and were vacuum dried while gradually warming to room temperature. After acquiring optical microscope images, the dried sample tissues were subject to derivatization using a TM-Sprayer (HTX Technologies, Chapel Hill, NC, United States) or by a home-made electrospray deposition system similar to that of Wu et al. (2016). The TM-Sprayer conditions for the derivatization agent solutions were the following: 30°C spray nozzle temperature, 30 μL/min flow rate, 8 criss-cross and off-set passes, 1200 mm/min spray nozzle velocity. The optimal derivatization reagent concentrations were the following: 20 mg/mL CA (in methanol), 10 mg/mL GT (in methanol with 2% TFA), and 6 mM 2-PA with 30 mM of the activation reagents (TPP and DPDS) (all in acetonitrile). For the electrospray deposition, +5 kV was applied to the spray nozzle with the tissue-attached slide held at ground at a flow rate of 33 μL/min and the emitter-to-tissue distance of ∼3 cm.

A 6.5 mM potassium acetate solution was sprayed with a TM-Sprayer prior to matrix deposition using the same condition for derivatization, to improve ion signals for potassium ion adducts. Then, the matrix was deposited by sputter coating (108 Auto Sputter Coater, Ted Pella Inc., Redding, CA, United States) gold at 40 mA for 20 s, or by spraying 40 mg/mL DHB (70% Methanol) or 20 mg/mL DAN (in acetonitrile) with TM-Sprayer. The TM-Sprayer conditions for the organic matrices were the following: 75°C spray nozzle temperature, 100 μL/min flow rate, 8 criss-cross and off-set passes, and 1200 mm/min spray nozzle velocity.

### Mass Spectrometry Imaging Analysis

Mass spectrometry imaging data were collected using a MALDI-linear ion trap-Orbitrap mass spectrometer (MALDI-LTQ-Orbitrap Discovery; Thermo Finnigan, San Jose, CA, United States). The instrument was modified to incorporate an external 355 nm frequency tripled Nd:YAG laser (UVFQ; Elforlight, Daventry, United Kingdom) and a f=60 mm focus lens (Feenstra et al., 2017b) using a 5× beam expander to reduce the laser spot size to <10 μm. TunePlus and XCalibur (Thermo Fisher Scientific) were used to define imaging parameters and to acquire data, respectively. Maize leaves and roots were acquired using 10 μm raster step size. Mass spectra were acquired with 10 laser shots per spectrum mostly using an Orbitrap mass analyzer (resolution of 30,000 at *m/z* 400) for an *m/z* scan range of 100–1000. Data were all obtained in positive mode with DHB (for GT derivatization) and gold (for CA and 2-PA derivatization) as matrices, except for fatty acid comparison shown in Supplementary Material which is also obtained in negative mode with DAN. MS/MS was performed for selected compounds on an adjacent tissue section with ion-trap analyzer using a mass tolerance of ±1.0 Da and normalized collision energy of 35.

MS images were generated using ImageQuest (Thermo Fisher Scientific) with a mass window of ±0.003 Da. Peak assignments were based on accurate mass measurement, tandem MS, and comparison with online databases (METLIN^1^ and Maize Genetic and Genomics Database^2^). Signal intensities for derivatized compounds were extracted from each maize cross-section sample (3 replicates of each genotype/tissue type, 36 total samples) and then the tentatively assigned metabolite features are uploaded to MetaboAnalyst^3^ (Chong et al., 2018) for statistical analysis. The following MetaboAnalyst settings were used for multivariate statistics: (i) data type: peak intensity table, (ii) no data filtering, (iii) no sample normalization, data transforming or data scaling.

### GC-MS Analysis of Total Metabolite Extraction

Total metabolites were extracted from maize leaves and roots (inbred B73 and Mo17; three replicates for each genotype and tissue type) as described by the Metabolomics Standards Initiative (MSI Board Member, Sansone et al., 2007). All reagents were ultra-pure (>99%) and were purchased from Millipore-Sigma (St. Louis, MO, United States). Samples were prepared as described in Noutsos et al. (2015) with minor modifications. Prior to metabolite extraction, plant samples (weighed and ground with liquid nitrogen) were spiked with 25 μg of ribitol and 20 μg of nonadecanoic acid (internal standards). The samples were homogenized with 0.35 mL methanol at 60°C. The mixture was immediately incubated for 10 min at 60°C and then sonicated for 10 min. Chloroform (0.3 mL) and water (0.3 mL) was added, and the mixture was vortexed for 5 min. After centrifugation to separate phases, the upper polar phase and the lower non-polar phase were separated. The polar fraction and non-polar fractions were dried using a speed vacufuge.

The extracts were methoximated with methoxyamine hydrochloride at 30°C for 90 min, followed by silylation with N,O-bis(trimethylsilyl)trifluoroacetamide (BSTFA)/trimethylchlorosilane (TMCS) at 60°C for 30 min, and then subjected to GC-MS analysis on Agilent 6890N gas chromatograph with 5973 Agilent MSD. The separation column was an Agilent HP5MSI (30 m × 0.250 mm, 0.25 μm). The oven program was as follows: initial temperature of 70°C for 2 min followed by 5°C/min ramp to 320°C, hold for 5 min. The mass range was set to scan *m/z* 40–900. The GC-MS was controlled by the Agilent ChemStation software. Metabolite identification was performed by comparing the mass spectra to the NIST14 Library.

## Results and Discussion

### Finding the Optimal Experimental Conditions for Derivatization Reactions

**FIGURE 1:**
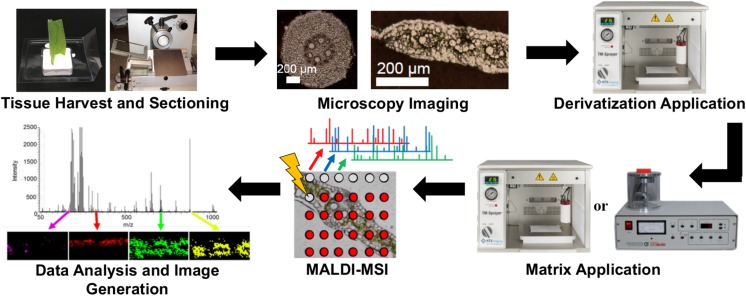
Overall workflow for MALDI-MSI with on-tissue derivatization.

**SCHEME 1:**
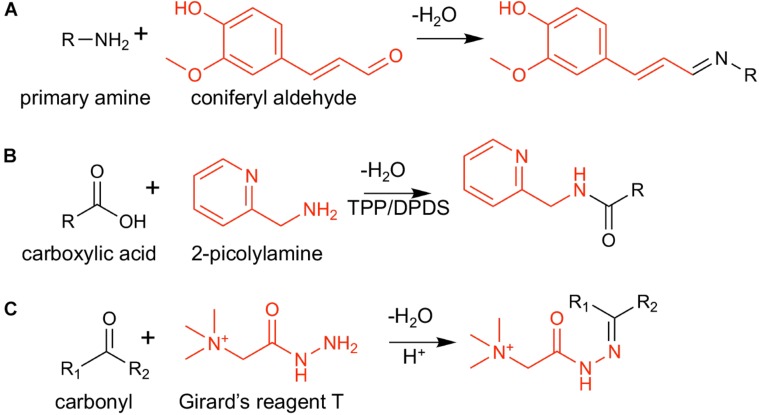
On-tissue chemical derivatization reactions used in this study: **(A)** coniferyl aldehyde (CA) and a primary amine, **(B)** 2-picolylamine (2-PA), and a carboxylic acid, and **(C)** Girard’s reagent T (GT) and a carbonyl group. Derivatization reagents are shown in red.

The overall workflow is shown in Figure 1. Tissues were harvested, cryo-sectioned, and vacuum dried. After acquiring optical microscope images, the dried tissues were subjected to chemical derivatization and matrix deposition prior to MALDI-MSI analysis. Reactions to chemically modify three functional groups are shown in Scheme 1. The combination of these three functional groups is expected to cover a majority of metabolite compounds with enhanced ionization efficiency in positive ion mode. For example, a commercially available large metabolite library (MSMLS, Mass Spectrometry Metabolite Library of Standards; IROS Technology, Bolton, MA, United States) contains 634 common metabolic compounds: 211 of them contain primary amines, 262 contain carbonyl groups, and 292 contain carboxylic acids. Out of the 88 compounds that do not have any of the three chemical functionalities, most of them have multiple alcohol groups (i.e., carbohydrates) which can be easily ionized as an alkaline ion adduct without the need of derivatization. This derivatization strategy was optimized and applied to maize tissue cross-sections, for which we have previously demonstrated cellular heterogeneity in single cell resolution but only for abundant metabolites such as lipids, phenolics, benzoxazinone derivatives, sugars, phosphate sugars, flavonoids, and flavonoid glycosides (Korte et al., 2015; Dueñas et al., 2017).

**FIGURE 2:**
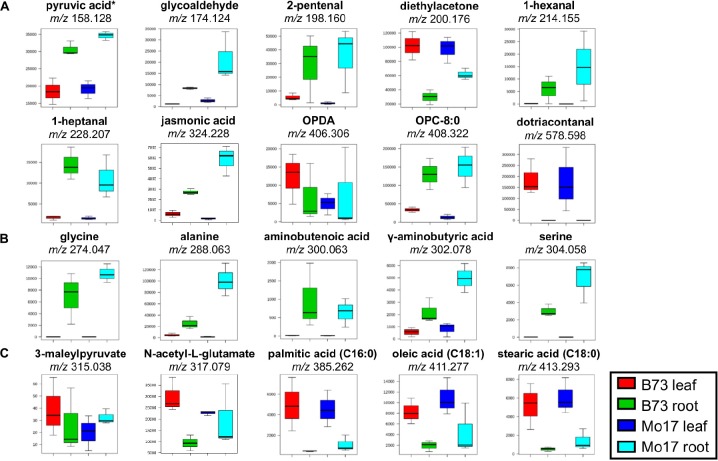
Box and whisker plots for selected metabolites derivatized with **(A)** GT, **(B)** CA, and **(C)** 2-PA. ^∗^Pyruvic acid is a fragment with CO_2_-loss as discussed in the text. Only one example is shown out of all the possible metabolites; see Supplementary Table S1A for other possible metabolites. All data is obtained in positive mode.

The experimental conditions such as concentration of derivatization reagent, TM-sprayer flow rate, and number of passes in the TM-sprayer, were optimized based on the number of new unique features observed only on the derivatized tissues. The derivatization reactions have been previously published and are known to be reproducible. Moreover, the TM-Sprayer used to deposit the derivatization reagents allows to control multiple parameters to guarantee reproducibility between samples. To illustrate this point, Supplementary Figure S1 shows the reproducibility of MS images of two consecutive cross-sections. Due to limited reaction time, we do not expect that all functional groups present in the sample would be derivatized, nor that all the metabolites present would be fully derivatized. Yet, we expect sample to sample variation is minimal as long as all the experimental procedures are kept robust, as apparent from the minimal standard deviations for many compounds in Figure 2.

Three levels of filtering were applied to extract those peaks that are unique and not present in the underivatized tissue sections. In the first filtering, any *m/z* values at noise level with an absolute intensity below 500 (signal-to-noise ratio of 20) were removed. In the second filtering, known contamination peaks were removed, as well as alkaline ion adducts and ^13^C isotope peaks using the mass tolerance of 5 ppm. Lastly, MS images were generated for all remaining *m/z* values using MSiReader (Robichaud et al., 2013), and inspected to ensure they were not present in the underivatized sample or non-tissue area. The mass difference due to the derivatization (Δm_der_) is 160.052, 114.103, and 90.058 Da for CA, GT, and 2-PA, respectively. GT modified compounds are detected as [M+Δm_der_]^+^, while CA or 2-PA modified compounds are mostly detected as potassium ion adduct, [M+Δm_*der*_ +K]^+^.

Maize root cross-sections were used to find effective matrices and optimize TM-Sprayer conditions for derivatization reactions. Based on our previous experience (Hansen et al., 2018a), DHB and sputter coated gold were selected to be tested as potential matrices. Sputtered Au has been used to analyze fatty acids and triacylglycerol species on tissues (Tang et al., 2011; Dufresne et al., 2016), and to provide effective ion signals across a broad range of metabolites in positive ion mode (Hansen et al., 2018a). DHB is well known to be effective for a relatively wide variety of compounds including lipids, oligosaccharides, organic acids, sugars, flavonoids, and their conjugates (Gemperline et al., 2014). Dozens of new peaks were detected with the on-tissue derivatization reactions. With DHB as a matrix, 25, 85, and 70 unique metabolite features were detected for the derivatization with CA, GT, and 2-PA, respectively. Using gold, 151, 53, and 108 unique features were detected, respectively. Gold was selected as the optimal matrix for CA or 2-PA derivatized compounds, and DHB was selected as the optimal matrix for GT derivatized compounds.

While over 100 new unique features were detected with CA and 2-PA as derivatization agents and gold as a matrix, the features observed using GT were limited, only 53 with gold and 85 with DHB. Electrospray (ESI) deposition of reagent is known to enhance derivatization efficiency for certain classes of compounds (Badu-Tawiah et al., 2012; Wu et al., 2016). In particular, microdroplet reaction acceleration is reported in the detection of cortisone using reactive DESI with GT (Badu-Tawiah et al., 2012). Unlike the other two reactions, GT is an acid-catalyzed reaction (Scheme 1C), and the reaction acceleration is attributed to the low pH of the ESI microdroplets. When using electrospray deposition, the number of unique features observed for the GT derivatization reaction is dramatically increased to 365 with DHB matrix, fourfold than neutral droplets using TM Sprayer. In contrast, there was no improvement for CA and 2-PA when they are ESI deposited with the number of unique features of 77 and 101, respectively, using gold matrix. The numbers of newly identified metabolite features with the derivatization are comparable to those without derivatization. Before the derivatization, the numbers of metabolite features are 211 and 114 from leaves with gold and DHB, respectively, and 396 and 221 from roots with gold and DHB, respectively.

### Statistical Analysis of Different Genotypes and Tissue Types

This multiple on-tissue derivatization strategy was applied to cross-sections of roots and leaves of two distinct maize genotypes (B73 and Mo17). Inbred B73 is derived from the Stiff Stalk Synthetic population generated at Iowa State University, and Mo17 was selected from Lancaster Sure Crop material (Troyer, 2004). These inbreds differ significantly in their genomic structure and this genetic diversity has been translated into metabolic, physiological, and phenotypic differences (Munamava et al., 2004). This approach was used to explore the metabolic differences arising from the genetic and tissue-type differences between these two inbred.

The first step in this analysis was to tentatively assign peaks. Peak assignments were based on accurate mass measurement, and comparison with online databases (METLIN, and Maize Genetic and Genomics Database). Tentatively identified compounds are listed in Supplementary Table S1A and the mass list for all of the derivatized features are provided in Supplementary Tables S1B–D. It is important to note that since it is not possible to differentiate between isomers, all known metabolites with the given chemical composition in Maize Genetic and Genomics Database are listed as tentatively assigned in Supplementary Table S1A, if they have the corresponding functional groups. For example, compounds derivatized with GT, CA, or 2-PA, need to include a carbonyl group, primary amine, or a carboxylic acid, respectively.

Combining both leaf and root data sets, a total of 656 new unique metabolite features were detected with derivatization (excluding 9 overlapping ones): 397 for GT, 151 for CA, and 117 for 2-PA. This is in addition to 883 unique metabolite features that could be detected without derivatization with DHB and gold matrix from both tissues, resulting in over 1,500 total metabolite features. Overlapping peaks were determined by comparing the accurate masses of the unique features after subtracting their corresponding derivatized mass (Δm_der_) and the potassium ion mass for CA or 2-PA. No derivatized compounds with multiple modifications of the same functional groups were detected, unlike commonly found in silylation in GC-MS. We recognize potential side reactions and the reactivity difference between the compounds. Considering the fact that we only see nine compounds with dual functionality out of 656 compounds and no multiple derivatization of the same functional group, we believe the reaction efficiency is limited most likely due to limited reaction time in the on-tissue modification reactions. When microdroplets of reagent arrive on the tissue surface, it only takes one or two seconds before the solvent evaporates, during which the reaction should be completed. As such, we suspect side reactions are minimal, although further studies should be conducted.

Then, the differences in metabolite abundance between the tissue-types and inbreds were investigated. Quantification is known to be difficult in MS imaging although it has been demonstrated for a limited number of compounds using isotopically labeled internal standards. As the objective of this study is untargeted analysis in a global scale, it is not feasible to achieve full quantification of all the compounds. Therefore, relative quantification is used to explore the metabolic differences between the samples. For this analysis, an unsupervised statistical analysis method, principal component analysis (PCA), was used to simplify the complex data. From a large set of data, PCA finds features (i.e., *m/z* values) that increase the variance between different groups and decrease the variance between similar groups. PCA is commonly used in typical GC or LC-MS based metabolomics (Bajoub et al., 2016).

**FIGURE 3:**
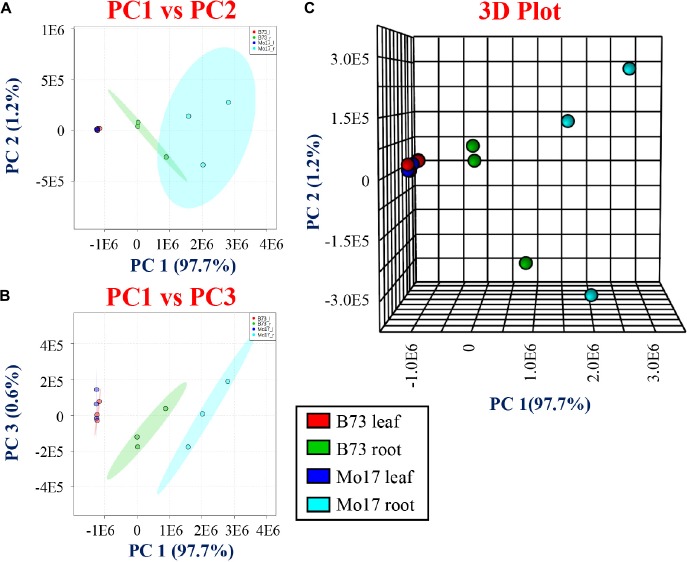
Principal component analysis (PCA) for derivatized metabolites using CA, 2-PA, and GT comparing the genotypes and tissues. **(A)** PC1 vs. PC2, **(B)** PC1 vs. PC3, and **(C)** three-dimensional PCA comparing two genotypes (B73 and Mo17) and tissue sections (roots and leaves).

The raw ion intensities of each tentatively assigned derivatized metabolite were extracted from each MALDI-MSI dataset using MSiReader (Robichaud et al., 2013). The intensities were then summed together across all the tissue area, and then averaged over the number of pixels of each tissue. A total of 36 data sets (three biological replicates, two genotypes, two tissue types, three derivatization reactions) were collected under the identical instrumental settings. The web-based metabolome process software, MetaboAnalyst 3.0, was utilized for multivariate statistical analysis to explore the differences between the roots and leaves and the two maize genotypes B73 and Mo17. Figure 3 shows two- and three-dimensional PCA plots showing PC1 vs. PC2 (Figure 3A), PC1 vs. PC3 (Figure 3B), and the three-dimensional PCA plot (Figure 3C). For leaf cross-sections, a separation is not clear between the different genotypes, B73 and Mo17, while a distinct separation is observed with the root cross-sections, especially when plotting PC1 with PC3 (Figure 3B). The three-dimensional PCA plot can separate the different genotypes nicely and the root genotypes, as shown in Figure 3C.

Box and whisker plots (Figure 2) were constructed by plotting the relative intensities of selected tentatively assigned metabolites within each sample group. Pyruvic acid at *m/z* 158.129 in Figure 2A is initially assigned as acetaldehyde (C_2_H_4_O after subtracting Δm_der_ of 114.103 Da), but acetaldehyde is volatile (b.p. ∼20°C) and very unlikely to be present in high abundance in plant tissues other than in some fruits (Fidler, 1968). We hypothesized it might be pyruvic acid (C_3_H_4_O_3_) which is highly abundant in plant tissues due to its important role in glycolysis, but degraded to lose CO_2_ during MALDI process. To prove this hypothesis, pyruvic acid standard was spotted on a glass slide, derivatized with GT, and MALDI-MS and MS/MS spectra were acquired. As shown in Supplementary Figure S2a, *m/z* 158.128 is dominant in the MALDI-MS spectrum of derivatized pyruvic acid, and its MS/MS spectrum (Supplementary Figure S2b) is consistent with the MS/MS spectrum on the tissue (Supplementary Figure S2c). Intact derivatized pyruvic acid is present in MS of standard (Supplementary Figure S2a), and also found on the tissue although in low abundance. It should be pointed out that other α-keto acids may also be decarboxylated in the MALDI source.

Some metabolites shown in Figure 2 have a significantly higher abundance in the root tissue section compared to leaves, regardless of the genotype [amino acids (glycine, alanine, serine), pyruvic acid, 1-hexanal, 1-heptanal, jasmonic acid, etc.]. JA, a signaling molecule that regulates defense and development in plants, and OPC-8:0, a metabolite in the linoleic acid cascade leading to JA (Ainai et al., 2003), shows a higher abundance in roots regardless of genotypes. These metabolites are oxylipin derived from either enzymatic or autoxidation of free or membrane-esterified fatty acids (Borrego and Kolomiets, 2016). Their high abundances in roots versus leaves are in good agreement with Gao et al. (2007) who reported JA content of ∼0.1 nmol/g in B73 leaves compared to ∼0.3 nmol/g in roots in comparison to 3–5 times of JA observed in roots vs. leaves in our data (Figure 2A).

The high abundance of amino acids in roots (Figure 2B) is due to the fact that they are stored in the root and then transported to other parts of the plant (Okumoto and Pilot, 2011). Amino acids are considered the main nitrogen carriers, are localized in specialized cells, and then transported through vascular systems when needed. For example, glutamine (primary nitrogen assimilation) is used to synthesize other amino acids and nitrogen-rich compounds by transamination, a process that occurs in the root tissue (Okumoto and Pilot, 2011). Once synthesized, amino acids are delivered to the leaves, and other sink organs such as flowers and seed. Furthermore, amino acids are observed in higher abundance in Mo17 than in B73, which is consistent with the previous reports (Römisch-Margl et al., 2010; Feenstra et al., 2017a). The GC-MS data of amino acids (Supplementary Figure S3) agrees well with the MALDI-MSI data shown in Figure 2, further validating the quantitative reliability of the derivatization reactions.

Conversely, some metabolites, such as several carboxylic acids (palmitic acid, oleic acid, linoleic acid) (Figure 2C) and dotriacontanal (Figure 2A), are present in much higher abundance in leaves than in roots. The high abundance of fatty acids in leaves is in agreement with Chaffai et al. (2007) who reported the total fatty acids as 5.9 ± 1.3 mg g^–1^ in root versus 30.4 ± 4.0 mg g^–1^ in shoot, similar to 3–6 times higher abundance in leaf vs. root for palmitic, oleic, and linoleic in our data. Dotriacontanal (C32:0 aldehyde) is a cuticular wax lipid which not only provides the first barrier protection against UV-radiation and fungi, but also plays a key role in preventing non-stomatal water loss (Hansjakob et al., 2011). Cuticular lipids are the first contact between an airborne pathogen and highly enriched on the surface of leaf tissues.

### High-Spatial Resolution Mass Spectrometry Images

Maize roots have a unique architecture which allows for an efficient uptake of water and nutrients and provides anchorage (Hochholdinger, 2009). Bright-field microscope images and anatomical assignments are shown in Supplementary Figure S4 for the cross-section of a B73 root. Maize roots exhibit a central vascular cylinder composed of the pith, xylem vessels (the largest vascular elements that are responsible for the transport of nutrients and water), and the pericycle (the outermost cell layer of the inner cylinder). The ground tissue is made up of a single endodermis layer, multiple layers of cortex tissue and single epidermis cell layer. Maize leaves exhibit the characteristic C4 Kranz anatomy (Haberlandt, 1882; Brown, 1975) in which concentric rings of bundle sheath cells encircle closely spaced veins (vascular bundles) and are surrounded by mesophyll cells (Supplementary Figure S4).

**FIGURE 4:**
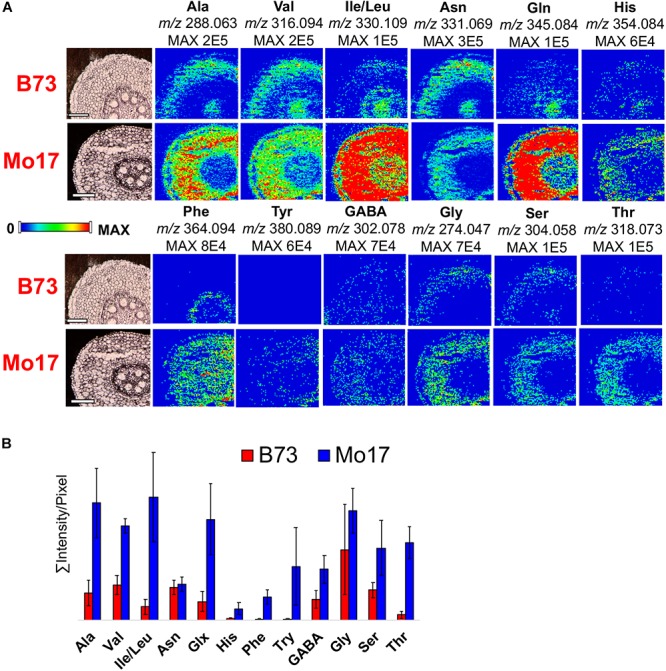
**(A)** MS images and **(B)** bar graph comparing twelve amino acids in the root cross-sections of B73 and Mo17. All data is obtained in positive mode.

Out of over five hundred unique features that are visualized with the chemical modifications, MS images were constructed for selected compounds, covering various classes of compounds. As shown in Supplementary Figure S5, 2-PA derivatized free fatty acids were clearly detected in positive ion mode whereas no signal was detected without derivatization. In negative ion mode, free fatty acids are detected in similar or higher ion signals compared to the derivatized ion signals in positive mode, but suffer from significant background contamination coming from vacuum pump oils. MS images of twelve CA derivatized amino acids were compared between the two genotypes in Figure 4. All amino acids are observed in higher abundance in the roots of Mo17 than B73, which is not surprising especially in the early phase of maize root development considering the Mo17 seeds contain much higher content of amino acids (Römisch-Margl et al., 2010; Feenstra et al., 2017a). Interestingly, amino acids show different localization between the two inbred. Amino acids are present almost exclusively in cortex for Mo17 but most amino acids are also present in pith for B73. To verify this genotype-dependent localization difference is not due to the artifact originated from ion suppression, a validation experiment was performed by spraying alanine-d4 on two root samples (B73 and Mo17). Selected amino acid images obtained by normalization to derivatized alanine-d4 (*m/z* 292.088) in Supplementary Figure S6 show consistent result, significant ion signals in pith only for B73. Currently, we do not have a good explanation for this genotype-dependent differential localization. One possible hypothesis is that some amino acids (i.e., Ala, Val, Ile/Leu, etc.) are mainly supplied from the shoot through the vascular transport in B73, while Mo17 can synthesize these amino acids in the root cortex.

**FIGURE 5:**
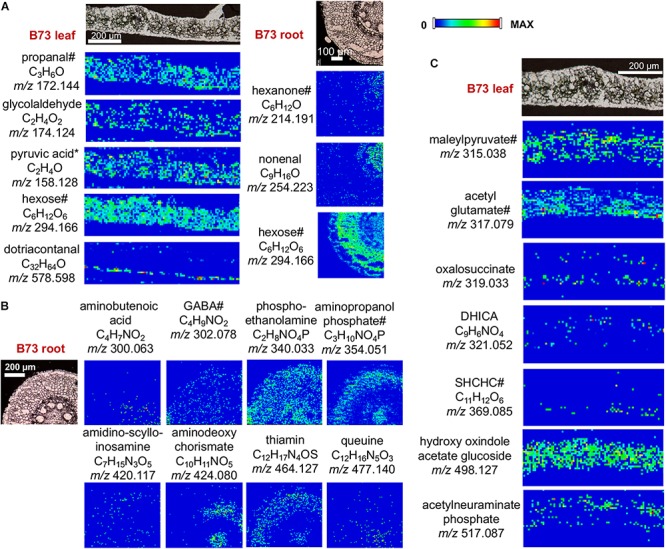
MS images of selected metabolites derivatized with **(A)** GT, **(B)** CA, and **(C)** 2-PA. DHICA, dihydroxy indole carboxylic acid; SHCHC, hydroxyl succinylcyclohexadiene carboxylate. Pyruvic acid is a fragment with CO_2_-loss as discussed in the text. ^#^Only one example is shown out of all the possible metabolites; see Supplementary Table S1A for other possible metabolites. All data is obtained in positive mode.

Selected images of other compounds detected by the derivatization with CA, GT, and 2-PA are shown in Figure 5. MS/MS was conducted on selected metabolites to assist compound identification as shown in Supplementary Figure S7. In most cases, however, MS/MS spectra are dominated by fragmentation from the derivatization agents and do not provide useful structural information of metabolites, although it does confirm they are derivatized compounds and their relative intensities might partially support their structures.

The images are shown only for B73, as there is no significant difference for these metabolites in Mo17. As observed in Figure 5, these metabolites show characteristic distributions in the tissue. For example, aminobutyric acid and phospho-ethanolamine (Figure 5A) are homogeneously distributed throughout the entire tissue with exception of the xylem. On the contrary, dotriacontanal (Figure 5B) is localized in the epidermis layer as wax lipids (Hansjakob et al., 2011). Other metabolites in leaves (i.e., 1-propanol, glycolaldehyde, maleylpyruvate) are absent in the vascular bundles (veins), while hexose and hydroxyl oxindole acetate glucoside is more homogenously localized, as it is also present in the veins. In roots, some metabolites are distributed in the cortex and pith area (hexose, aminopropanol phosphate, aminodeoxychorismate, and thiamin) while others are distributed homogenously within the tissue (aminobutyric acid). It is important to note that many of these metabolites, which are essential cellular metabolites, have not been previously visualized directly on tissue, therefore, the significance of the specific localizations needs to be further explored.

## Conclusion

In this work, we adopted several on-tissue chemical derivatization reactions in order to facilitate the visualization of metabolites in an untargeted manner. Incorporating on-tissue chemical derivatization as an untargeted approach enabled the identification of over six hundred new metabolite features. Moreover, statistical analysis revealed the variation between metabolites in different tissue sections and between the different genotypes. This proof of concept experiment suggests that MALDI-MSI in the metabolomics scale is possible. A combination of derivatizations can be used on-tissue to overcome the inherent limitations of samples to allow for more complete metabolite visualization and detection in a single analysis.

Despite the growing popularity of MS-based metabolomics, key challenges remain to be resolved. The current significant challenges are the lack of adequate MS and MS/MS databases and minimizing any side reactions and/or unwanted ion suppression from the derivatization agents. Improvements to databases and sample preparation would further the metabolic coverage of this method. Moreover, adequate evaluation of method performance and validation is needed and further studies are in progress but are beyond the scope of the current work. Regardless of these limitations, we envision that the proposed approach of using on-tissue chemical modifications will not only improves the existing MALDI-MS technologies, but can also be used to advance toward the single-cell metabolomics scale.

## Data Availability

All datasets generated for this study are included in the manuscript and/or the Supplementary Files.

## Author Contributions

MD and YL designed the experiments. MD prepared the samples and conducted the experiments. MD, EL, and YL carried out the data analysis and wrote the manuscript.

## Conflict of Interest Statement

The authors declare that the research was conducted in the absence of any commercial or financial relationships that could be construed as a potential conflict of interest.

## Footnotes

1 https://metlin.scripps.edu

2 https://corncyc-b73-v4.maizegdb.org

3 http://metaboanalyst.ca

## Abbreviations

2-PA: 2-picolylamine
AP-SMALDI: atmospheric pressure scanning microprobe MALDI
CA: 4-hydroxy-3-methoxycinnamaldehyde
DAN: 1,5-diaminonaphthalene
DHB: 2,5-dihydroxybenzoic acid
DPDS: 2,2-dipyridyl disulfide
GC: gas chromatography
GT: Girard’s reagent T
JA: jasmonic acid
LC: liquid chromatography
MALDI-MSI: matrix-assisted laser desorption/ionization mass spectrometry imaging
MS/MS: tandem mass spectrometry
nano-DESI: nanospray desorption electrospray ionization
PCA: principal component analysis
tg-LAESI: transmission geometry laser ablation electrospray ionization
TPP: triphenylphosphine.
