# Corrigendum: Towards Mass Spectrometry Imaging in the Metabolomics Scale: Increasing Metabolic Coverage Through Multiple On-Tissue Chemical Modifications

**DOI:** 10.3389/fpls.2019.01079

**Published:** 2019-09-11

**Authors:** Maria Emilia Dueñas, Evan A. Larson, Young Jin Lee

**Affiliations:** Department of Chemistry, Iowa State University, Ames, IA, United States

**Keywords:** mass spectrometry imaging, metabolomics, on-tissue derivatization, high-spatial resolution, maize, single cell

In the original article, there was a mistake in the legend for **Figure 2** and **Figure 3** as published. The legends for **Figures 2** and **3** are switched. The corrected legend appears below.

**Figure 2:**
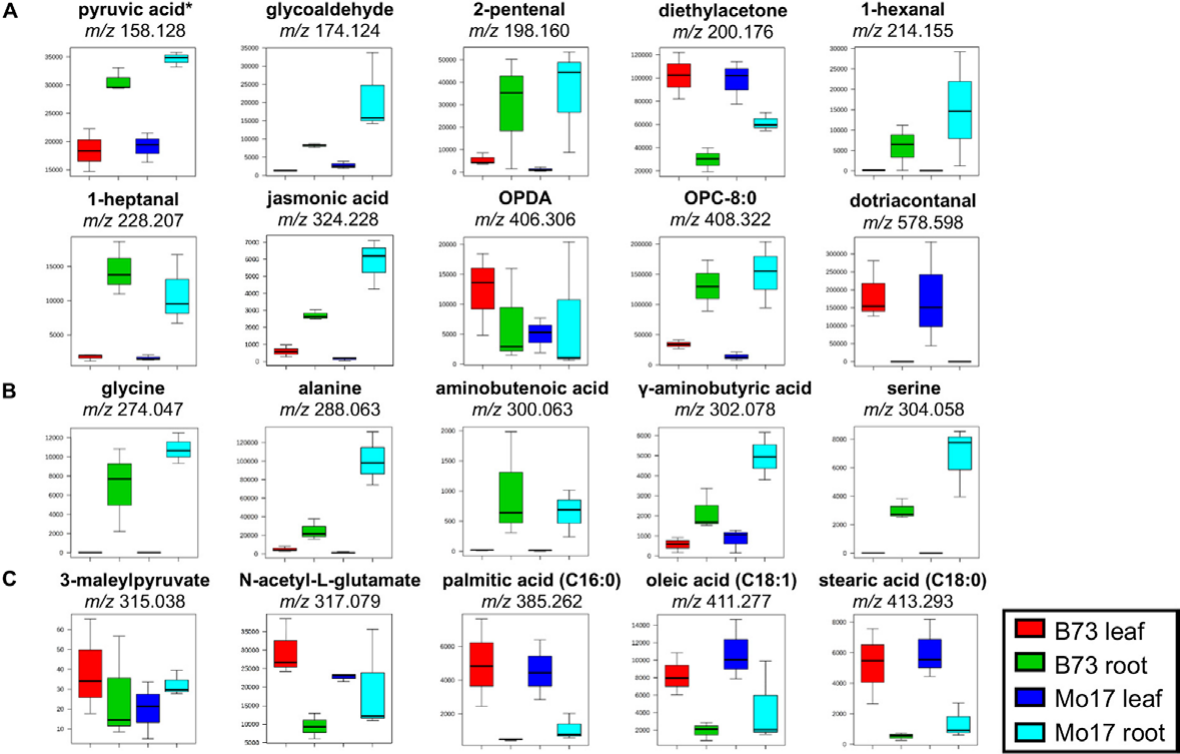
Box and whisker plots for selected metabolites derivatized with **(A)** GT, **(B)** CA, and **(C)** 2-PA. *Pyruvic acid is a fragment with CO2-loss as discussed in the text. Only one example is shown out of all the possible metabolites; see **Supplementary Table S1A** for other possible metabolites. All data is obtained in positive mode.

**Figure 3:**
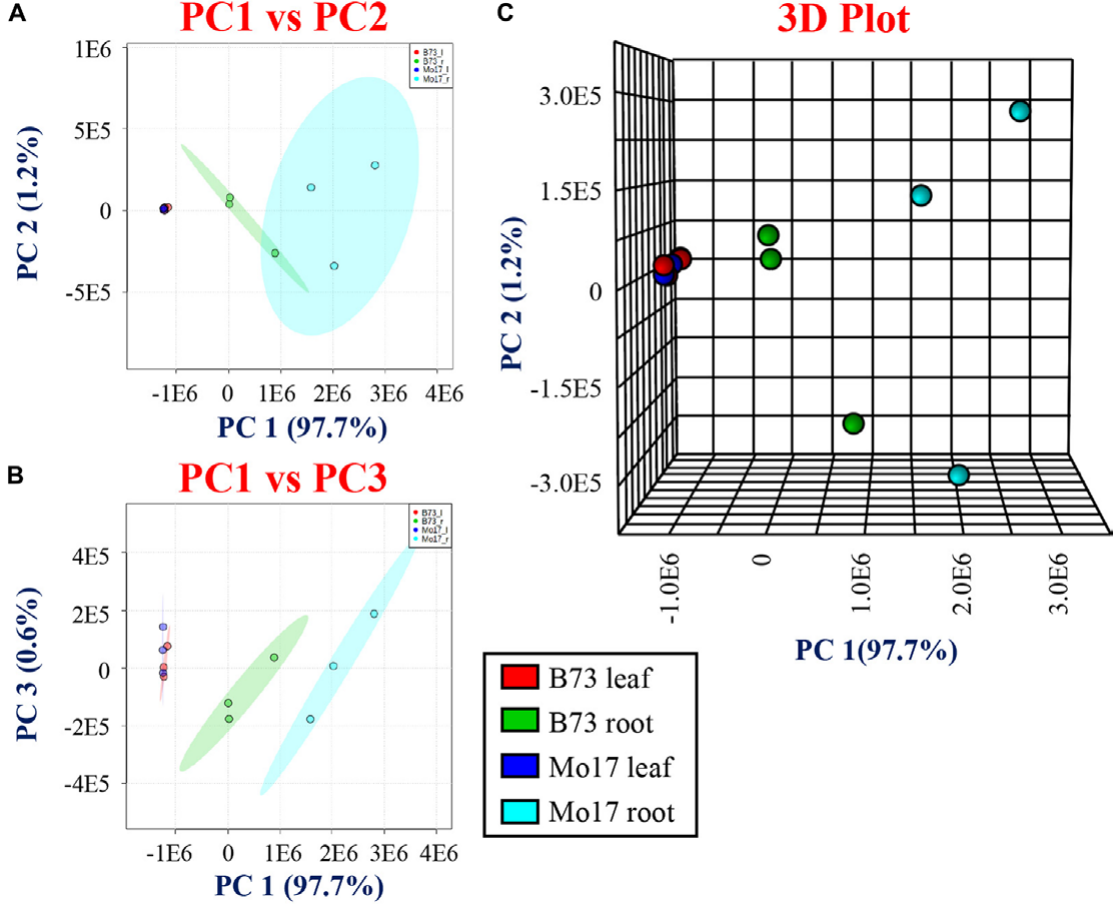
Principal component analysis (PCA) for derivatized metabolites using CA, 2-PA, and GT comparing the genotypes and tissues. **(A)** PC1 vs. PC2, **(B)** PC1 vs. PC3, and **(C)** three-dimensional PCA comparing two genotypes (B73 and Mo17) and tissue sections (roots and leaves).

The authors apologize for this error and state that this does not change the scientific conclusions of the article in any way. The original article has been updated.

