# Supplementary material for: Toward Mass Spectrometry Imaging in the Metabolomics Scale: Increasing Metabolic Coverage Through Multiple On-Tissue Chemical Modifications
Source: Front Plant Sci. 2019 Jul 12;10:860. doi: 10.3389/fpls.2019.00860 (PMC6639774; doi:10.3389/fpls.2019.00860)
Supplement: Supplementary file 2 [file Data_Sheet_1.pdf]

Supplementary Material

1 Supplementary Figures

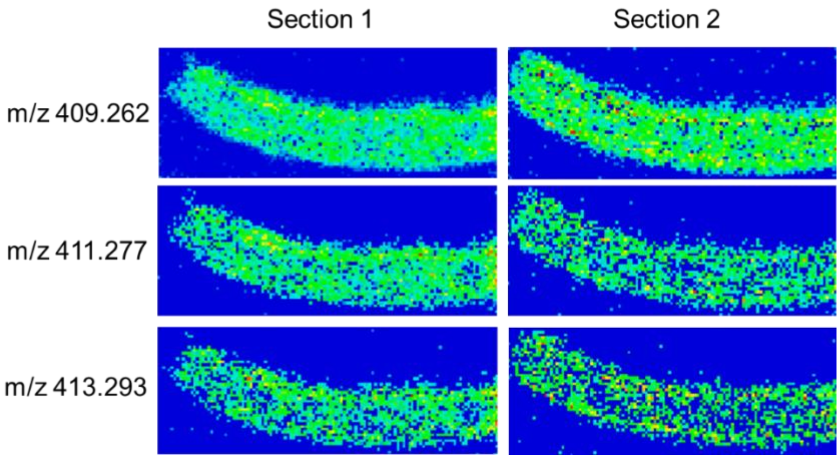

Supplementary Figure S1. Reproducibility between two consecutive using 2-picolylamine.

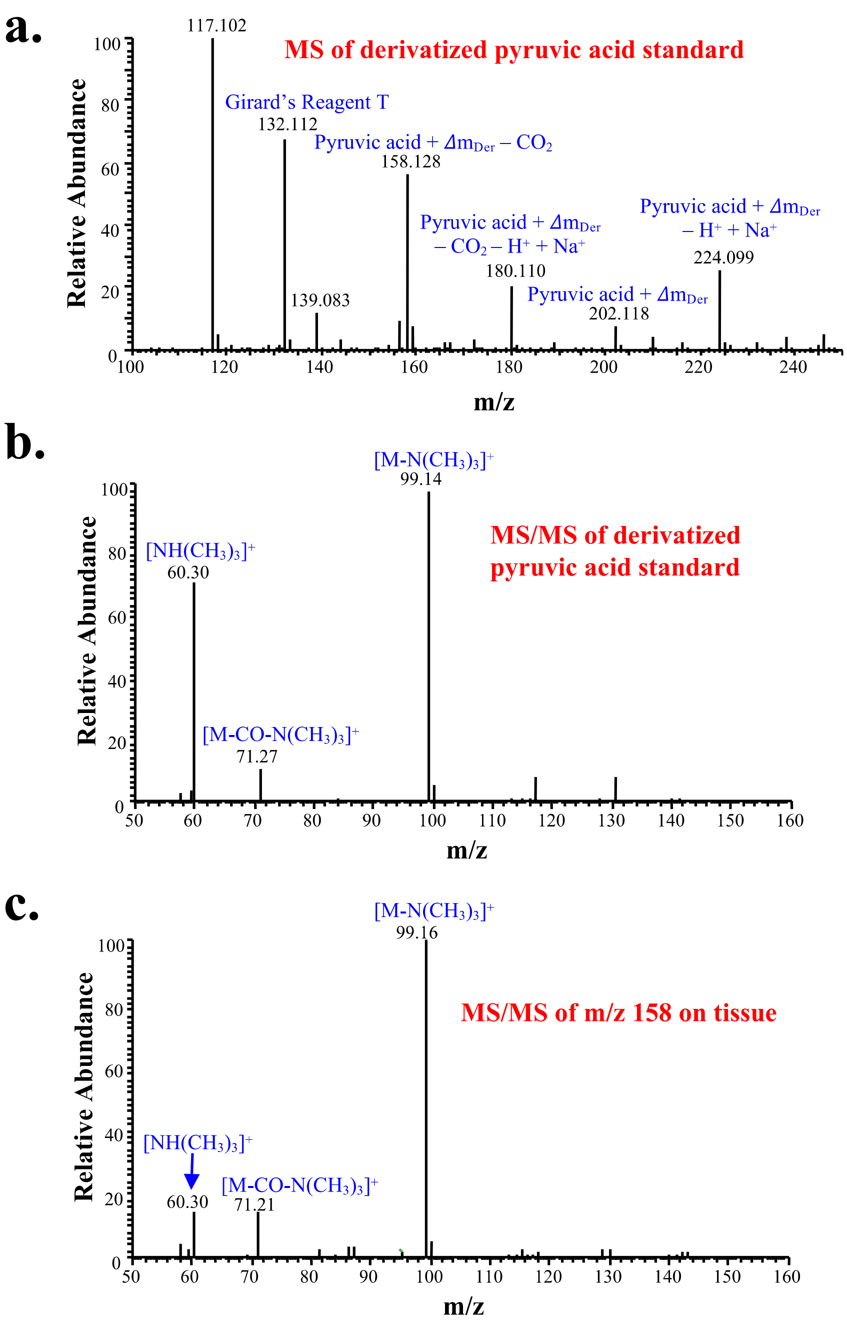

Supplementary Figure S2. (a) MS and **(b)** MS/MS of derivatized standard pyruvic acid, and **(c)** MS/MS of *m/z* 158.128 on tissue.

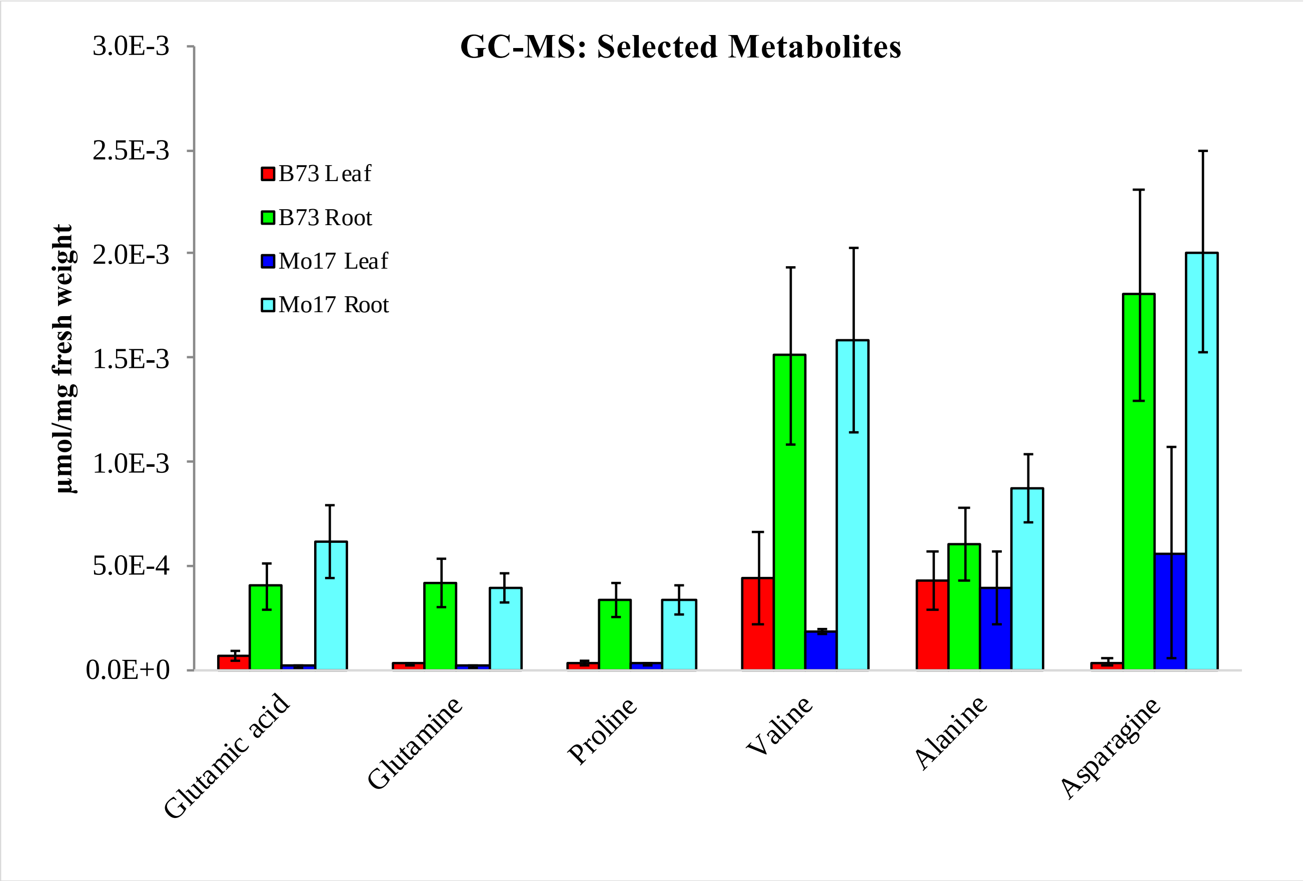

Supplementary Figure S3. GC-MS data for selected amino acids. These metabolites are detected as doubly or triply trimethylsilyl derivative. Three samples were used for each genotype and tissue type, with each sample consisting of several leaves or roots.

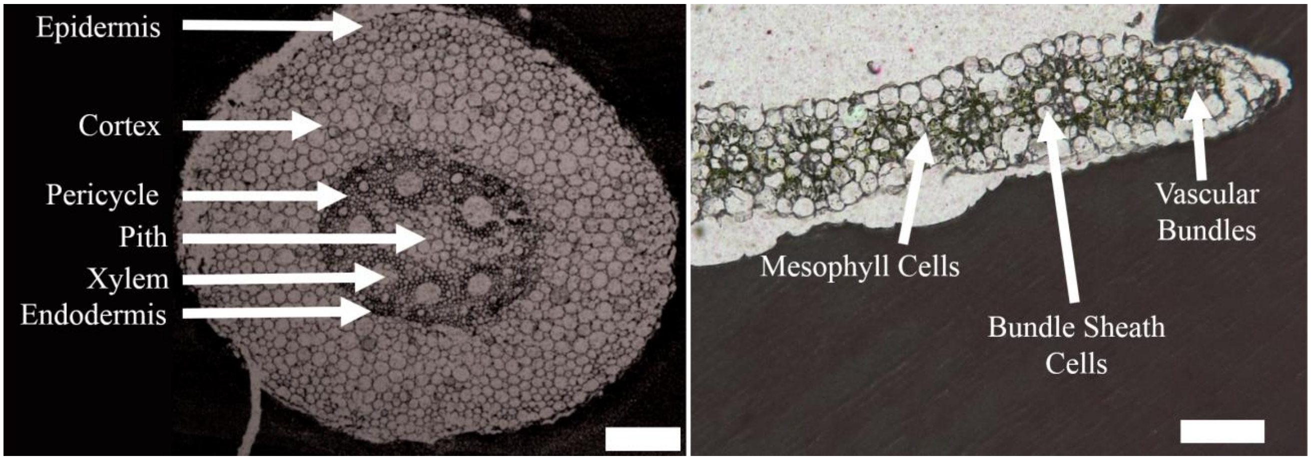

Supplementary Figure S4. Optical images on the cross-sections of a B73 maize root (left) and leaf (right) with anatomical annotations. Scale bar are 200 µm and 100 µm, for root and leaf, respectively.

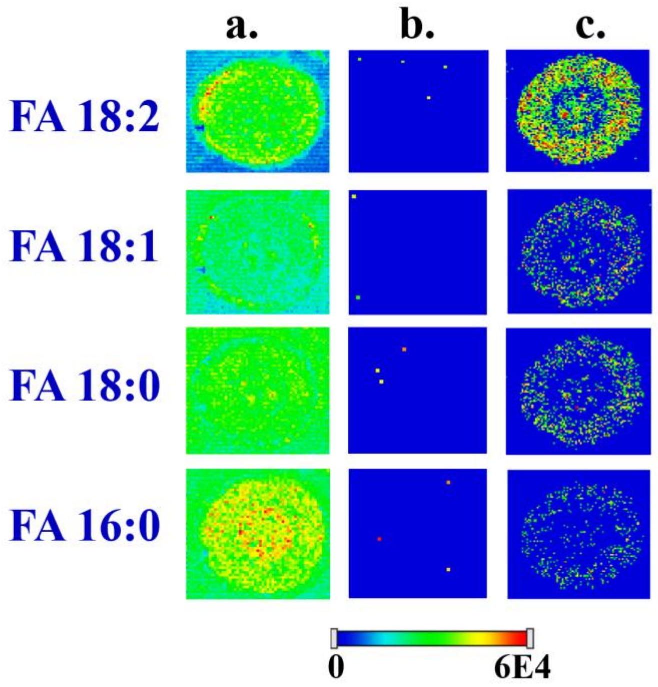

Supplementary Figure S5. MS images of free fatty acids for the cross section of maize root **(a)** without derivatization in negative ion mode using DAN as a matrix and detected as $[M\text{-}H]^-$, **(b)** without derivatization in positive ion mode using Au as a matrix and detected as $[M+K]^+$, and **(c)** with 2-PA derivatization in positive ion mode using Au as a matrix and detected as $[M+\Delta m_{der}+K]^+$. Δm_{der} = 90.058 Da.

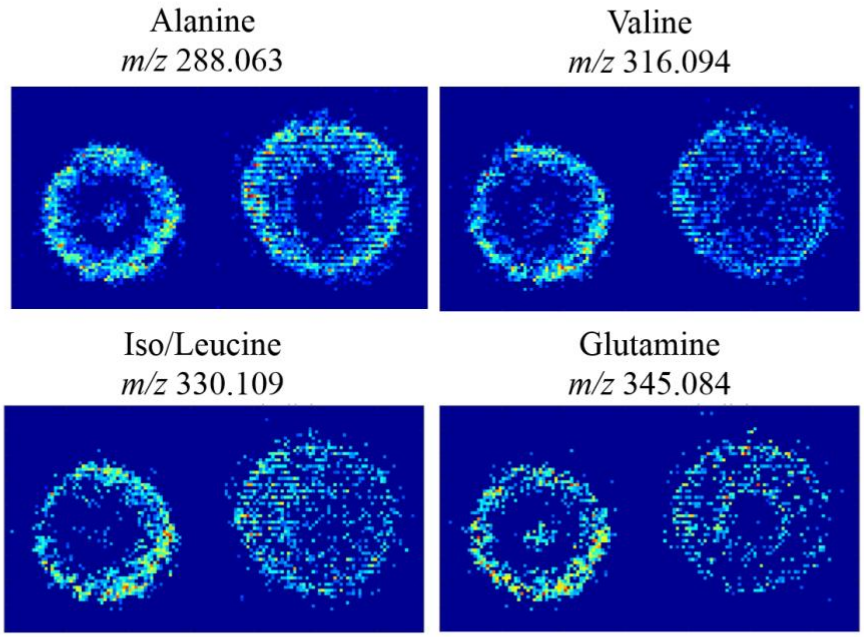

Supplemental Figure S6. MS images of roots (left-B73 and right-Mo17) for selected amino acids derivatized with CA. Alanine-d4 is sprayed on the tissue before the derivatization and all images are normalized to derivatized alanine-d4 (*m/z* 292.088).

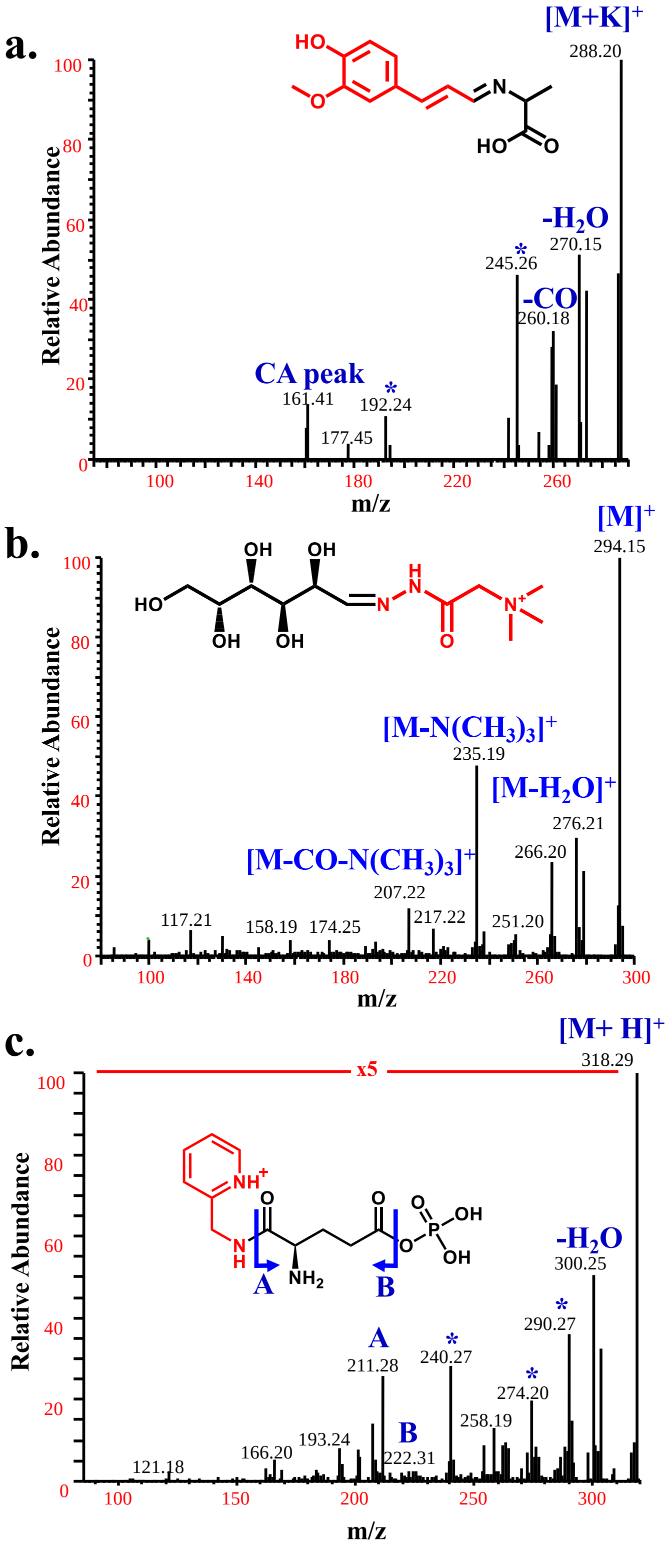

Supplementary Figure S7. Representative MS/MS spectra for selected compounds derivatized using **(a)** CA (alanine, *m/z* 288.20), **(b)** GT (aldehydogalactose, *m/z* 294.166), and **(c)** 2-PA (glutamyl-5-phosphate, *m/z* 318.083). Asterisks represent contaminations from co-isolated chemical contaminant.
